# Three-way interaction effects of early life stress, positive parenting and *FKBP5* in the development of depressive symptoms in a general population

**DOI:** 10.1007/s00702-021-02405-0

**Published:** 2021-08-22

**Authors:** Rebecka Keijser, Susanne Olofsdotter, Kent W. Nilsson, Cecilia Åslund

**Affiliations:** 1 Department of Neuroscience, Uppsala University, Uppsala, Sweden; 2 Centre for Clinical Research, Uppsala University, Västmanland County Hospital Västerås, 721 89 Västerås, Sweden; 3 School of Health, Care and Social Welfare, Mälardalen University, Västerås, Sweden; 4 Department of Public Health and Caring Sciences, Uppsala University, Uppsala, Sweden

**Keywords:** Adolescence, cG × E, Diathesis stress, Differential susceptibility, FKBP Prolyl Isomerase 5

## Abstract

*FKBP5* gene–environment interaction (cG × E) studies have shown diverse results, some indicating significant interaction effects between the gene and environmental stressors on depression, while others lack such results. Moreover, *FKBP5* has a potential role in the diathesis stress and differential susceptibility theorem. The aim of the present study was to evaluate whether a cG × E interaction effect of *FKBP5* single-nucleotide polymorphisms (SNPs) or haplotype and early life stress (ELS) on depressive symptoms among young adults was moderated by a positive parenting style (PASCQ^pos^), through the frameworks of the diathesis stress and differential susceptibility theorem. Data were obtained from the Survey of Adolescent Life in Västmanland Cohort Study, including 1006 participants and their guardians. Data were collected during 2012, when the participants were 13 and 15 years old (Wave I: DNA), 2015, when participants were 16 and 18 years old (Wave II: PASCQ^pos^, depressive symptomology and ELS) and 2018, when participants were 19 and 21 years old (Wave III: depressive symptomology). Significant three-way interactions were found for the *FKBP5* SNPs *rs1360780*, *rs4713916*, *rs7748266* and *rs9394309*, moderated by ELS and PASCQ^pos^, on depressive symptoms among young adults. Diathesis stress patterns of interaction were observed for the *FKBP5* SNPs *rs1360780*, *rs4713916* and *rs9394309*, and differential susceptibility patterns of interaction were observed for the *FKBP5* SNP *rs7748266*. Findings emphasize the possible role of *FKBP5* in the development of depressive symptoms among young adults and contribute to the understanding of possible differential susceptibility effects of *FKBP5*.

**Supplementary Information:**

The online version contains supplementary material available at 10.1007/s00702-021-02405-0.

## Introduction

The genesis of depression emerges from various factors, such as genetic predisposition and the environment (Sullivan et al. 2000), and seems to vary across age groups (Kaufman et al. 2001), signifying a complexity over different pathways to the onset of depression at different stages of development (Kaufman et al. 2001). Although the prevalence among children is low, the incidence of depression rises substantially throughout adolescence (Green et al. 2005) and is predictive of a wide range of long-term psychosocial impairments, including recurrent depressive disorders during early adulthood (Aalto-Setälä et al. 2002; Hanlon et al. 2020). One of the most robust findings is an increase in its prevalence in women after puberty, where twice as many women as men suffer from depression (Hyde et al. 2008).

The family environment is a contributing factor to the onset and maintenance of mood disorders, a linkage that has been recognized since the 1980s (Burbach and Borduin 1986; Gerlsma et al. 1990; Gorostiaga et al. 2019). Schwartz et al. (2017) suggest that adolescent depression may be predicted by parenting in three ways: as a direct effect, mediated by biopsychosocial factors, or as a moderator of the relationship between other biopsychosocial factors with adolescent outcomes. Furthermore, Schwartz et al. (2017) reported that adolescents with parents who expressed higher levels of aggression, lower levels of positivity or responded in negative terms towards their children’s behaviours were at greater risk for the development of depression. Positive parenting or a lack thereof has been associated with the onset of major depressive disorder during adolescence (Chen et al. 2009; Schwartz et al. 2014). Longitudinal effects of a positive parenting style on depression have shown a decrease in symptoms (del Barrio et al. 2016; Keijser et al. 2020b). Even though the effects of parenting style seem to have a moderate effect on depression, parenting style might be beneficial to the well-being of adolescents (Gorostiaga et al. 2019).

Another aspect in the development of depression is excessive external stress, in particular early life stress (ELS) (Wang et al. 2020). ELS refers to stressful life events that occurred during childhood, such as emotional, psychological, or physical abuse and neglect. ELS can cause a prolonged period of stress and have a negative impact throughout life (Hanlon et al. 2020; Teicher and Samson 2013). Exposure to ELS may lead to the development of mental illness (Bernet and Stein 1999; Hanlon et al. 2020) and confer a risk for depression up to young adulthood (Hazel et al. 2008; Shapero et al. 2014). More severe ELS exposure involves a more robust, persistent and stronger effect on depression (Gillespie et al. 2009; Zannas and Binder 2014). Notably, the occurrence of ELS may be more important than the form, severity or duration (Briere and Jordan 2009). Furthermore, childhood abuse is commonly accompanied by multiple types of abuses (Vachon et al. 2015). Therefore, when evaluating one form of ELS, it can be expected that other forms of ELS will likely co-occur, and poly-victimization may be present (Fisher et al. 2015). The environmental factors parenting styles and ELS have both been linked to depression, individually and together (Carr et al. 2013; Garber 2006; LeMoult et al. 2019; Sanders et al. 2014; Schwartz et al. 2012). Moreover, individuals that are exposed to negative life events and have certain cognitive tendencies of evaluating the exposure, or its consequences, negatively are more likely to develop depressive symptoms than individuals without such cognitive tendencies (Abramson et al. 1989). That is, when a cognitive vulnerability and stress interact it may increase the likelihood of depressive symptoms (Abramson et al. 1989). It is furthermore suggested that vulnerable individuals may behave in a way that partially causes stressful life events and subsequently increase a risk of developing depression (Hammen 1991). Kercher and Rapee (2009) investigated cognitive vulnerability among adolescents and found greater depression scores among those with high cognitive vulnerability than those with low cognitive vulnerability. These results are similar to those reported by Johnson et al. (2012) that suggested a positive association between initial levels of depressive symptoms and initial levels of negative life events and that initial levels of depressive symptoms furthermore increased the risk for future negative life events.

Even if individual- and social factors seem to be associated with the occurrence of depression, their effects are not independent of genetically determined vulnerability (Goldberg 2001; Kercher and Rapee 2009). Individuals vulnerable to depression supposedly have an impaired stress response (Binder 2009; Hori et al. 2010). A dysregulation in the hypothalamic–pituitary–adrenal (HPA) axis has been suggested as an important pathogenetic factor regarding depressive disorders (Holsboer 1999, 2000; Pariante and Lightman 2008; Spencer and Deak 2017). Previous research studies have evaluated genes involved in the regulation of the HPA axis to explore the genetic and functional architecture underlying HPA dysregulation in depression, such as *FKBP5* (Binder 2009; Binder et al. 2004).

The *FKBP5* gene is located on the short arm of the human chromosome 6 (6p21.31), a region covering several single-nucleotide polymorphisms (SNPs) and codes for the FK506-binding protein 51 (*FKBP5*) (Binder et al. 2004). *FKBP5* is a co-chaperone to the heat shock protein 90, which regulates glucocorticoid-receptor sensitivity (Binder 2009; Grad and Picard 2007; Pratt and Toft 1997). Cortisol elicits *FKBP5* expression as it activates the glucocorticoid-response elements (Vermeer et al. 2003). Simultaneously, *FKBP5* binding to the glucocorticoid-receptor reduces attraction for cortisol and diminishes the amount of activated glucocorticoid-receptor translocation to the cell nucleus (Grad and Picard 2007; Wochnik et al. 2005). The HPA system has a role in the response to stress (Stephens and Wand 2012) and dysregulation in the HPA axis is linked to depression (Holsboer 1999, 2000; Pariante and Lightman 2008; Spencer and Deak 2017). Consequently, due to its function in the HPA axis regulation, common variants of *FKBP5* are thought to play a role in the development and relief of depressive symptoms.

The combination of candidate genes and the environment (cG × E) can be evaluated as interaction effects where environmental measures in combination with specific candidate genes may influence the variance in psychological traits, such as depression (Dick 2011). Not all individuals exposed to ELS or lack of positive parenting develop depression. These differences in vulnerability could be explained by biological factors and further explained by cG × E interaction effects (Musci et al. 2019; Zannas and Binder 2014). ELS and parenting style have been recognized as important environmental factors in a mental health context (Chen et al. 2015; Hankin et al. 2011; Peyrot et al. 2018). Binder et al. (2004) showed that variants of the *FKBP5* gene were associated with a heightened risk of developing depression when individuals had also been exposed to ELS. Wang et al. (2018) presented a significant association between ELS and the *FKBP5* SNP *rs1360780* in their meta-analysis, where they observed an increased risk of developing depression under childhood adversity. *FKBP5* has further been suggested to have a dual effect with positive environment. For example, appraisal among psychiatric patients (Cristóbal-Narváez et al. 2020) has shown an increase in positive responses to the treatment of psychiatric illness and depression (Binder et al. 2004; Dam et al. 2019), while other studies have shown contrary findings (Isaksson et al. 2016; Pérez-Pérez et al. 2018). Previous knowledge regarding the associations of parenting style, ELS and the *FKBP5* gene on depression provides a cogent rationale for evaluating cG × E interaction effects. There are different theoretical frameworks underlying the cG × E approach. The diathesis stress model has been adapted in the research field of depression since the 1980s (Bebbington 1987). The model proposes that stress may influence vulnerability to transforming a tendency toward mental illness into concrete psychopathology (Monroe and Simons 1991). This suggests that vulnerable individuals are at disproportionate risk of being affected unfavourably by harmful stressors or risk factors (Belsky and Pluess 2009). With regard to cG × E studies, the diathesis stress model is the conceptual framework that dominates the research field today. However, the diathesis stress model focus on risk factors and excludes the possible influence of positive environments. An alternative approach to the diathesis stress model is the differential susceptibility theory, which suggests that susceptible individuals, rather than being solely responsive to negative environments, are also responsive to positive environments, in a “for better and for worse” manner (Belsky et al. 2016; Belsky and Pluess 2009; Hartman and Belsky 2016).

*FKBP5* studies have shown tendencies of the diathesis stress and/or differential susceptibility effects in relation to psychiatric outcomes (Bevilacqua 2012; Scheuer et al. 2015; VanZomeren-Dohm et al. 2015; Xie et al. 2010; Zimmermann et al. 2011). However, only one previous study evaluated the environmental context of positive and negative aspects regarding differential susceptibility of the *FKBP5* in relation to depression and performed statistical tests to investigate the pattern of the cG × E effects (Pérez-Pérez et al. 2018). Although significant cG × E interactions were found for social anxiety and neuroticism, no effects of *FKBP5* were found in relation to depression (Pérez-Pérez et al. 2018). The study included a rather small non-clinical sample (*n* = 86) in a cross-sectional design (Pérez-Pérez et al. 2018), prompting the need for further studies. The present study aims to expand the current knowledge by investigating the differential susceptibility effects of *FKBP5* in relation to depressive symptoms using a larger study sample and a longitudinal approach. Given the known influence of parenting style and ELS in relation to depression, these environmental factors are of interest for investigating the possible differential susceptibility properties of *FKBP5*.

## Aim

The aim of the present study was to evaluate whether a cG × E interaction effect of *FKBP5* SNPs or haplotype and ELS on depressive symptoms among young adults was moderated by a positive parenting style, through the frameworks of the diathesis stress theory and differential susceptibility hypothesis.

## Methods

### Study sample

The Survey of Adolescent Life in Västmanland Cohort Study (SALVe Cohort) collects data from individuals born in either 1997 or 1999 in Västmanland, Sweden. The present study includes data from 2012 when participants were 13 and 15 years old (M_age_ 14.4, SD_age_ 1.03; Wave I: DNA), 2015 when participants were 16 and 18 years old (M_age_ 17.36, SD_age_ 1.04; Wave II: Depressive symptomology, parenting styles and ELS) and 2018 when participants were either 19 or 21 years old (M_age_ 20.19, SD_age_ 1.03; Wave III: Depressive symptomology).

Out of 4712 eligible adolescents, 1868 consented to participate in the SALVe Cohort during Wave I (response rate: 40%), 1541 participated during Wave II (response rate from Wave I: 82%) and 1176 participated during Wave III (response rate from Wave II: 76%).

In the present study, 28 participants were excluded for randomly incomplete answers in the study variables. Included in the current study sample were 1006 Caucasian young adults (634 women) who provided data in all three waves and 1006 caregivers who provided data in Wave II.

### Measurements

#### Depressive symptoms

Depressive symptoms were assessed using the Depression Self-Rating Scale (DSRS) (Sjoberg et al. 2006; Svanborg and Ekselius 2003) during Waves II (16–18 years) and III (19–21 years). The DSRS is a self-report questionnaire that consists of 14 items measuring depressive symptoms, with yes/no statements. The DSRS is based on the A-criterion for major depressive disorder from the Diagnostic and Statistical Manual of Mental Disorders IV (DSM-IV) (American Psychiatric Association 2000; Svanborg and Ekselius 2003). The following symptom categories occurring during the last 2 weeks are included in the DSRS: 1. Dysphoric mood/irritability; 2. Loss of interest or pleasure in most activities; 3. Sleep disturbances; 4. Weight loss or gain/appetite disturbances; 5. Psychomotor agitation or retardation; 6. Fatigue or loss of energy; 7. Feelings of worthlessness or guilt; 8. Concentration disturbances; and 9. Thoughts of suicide.

All symptoms per time point were clustered and then categorized into two separate continuous depressive symptom summation indices for Waves II and III (where no = 0, and yes = 1). Participants were able to score 0–9 units (number of reported symptom categories for depressive symptoms) because some criteria were assessed using two items but were only counted as one criterion (Sjoberg et al. 2006). Internal consistency for the DSRS at Waves II and III was Cronbach’s *α* = 0.827 and *α* = 0.865, respectively. A previous study of the DSRS used the Structured Clinical Interview for DSM-IV depression module (SCID-I) (First et al. 1998) as diagnostic standard and a cut-off of at least five depressive symptoms on the DSRS (i.e., meeting DSM-IV major depression A-criterion), and reported a sensitivity of 96% and a specificity of 59% for classification of major depressive disorder among adult psychiatric patients (Svanborg and Ekselius 2003).

#### Early life stress

ELS was assessed by the caregivers completing the Neuro Pattern Questionnaire–Pre-/postnatal Stress Questionnaire (NPQ–PSQ) (Hero 2013) during Wave II. The NPQ–PSQ is a retrospective self-report questionnaire assessing ELS through four dimensions: pregnancy (e.g., relationship status), birth (e.g., special mediation intervention after birth), childhood (e.g., did any of the following stressors exist? Such as massive conflict within the family?) and general information (e.g., estimation of income during childhood). The caregiver’s subjective evaluation of the total impact of experienced stress exposure during childhood was used as a measure of ELS by the summarizing question: “Consider all questions regarding the child’s childhood/upbringing. How stressful do you consider the child’s childhood to be on a scale from 1 to 10 (i.e., not stressful = 1 to highly stressful = 10)?” In contrast, a summation index of frequency of reported events was considered less suitable when targeting individually experienced stress levels because the experienced stress impact of each reported event will differ subjectively between individuals. The NPQ–PSQ was translated into Swedish by researchers in the SALVe cohort group in accordance with recommended procedures (Nelson 2007; Whitaker 2012). The NPQ–PSQ questionnaire is part of the Neuropattern, a translational tool to detect and treat stress pathology. NPQ–PSQ has shown adequate psychometric properties (Hellhammer et al. 2012; Hero et al. 2012).

#### Positive parenting

Perceived positive parenting style was assessed through the Parents as Social Context Questionnaire (PASCQ) (Skinner et al. 2005; Taylor and Francis 2017), Swedish version (Keijser et al. 2020a) during Wave II when the participants were 16 or 18 years old.

The PASCQ is a 24-item self-rating scale providing scores on six parenting styles over two dimensions: i.e., a positive dimension including parenting styles of warmth (e.g., “My parents let me know they love me”), structure (e.g., “If I ever have a problem, my parents help me to figure out what to do about it”) and autonomy support (e.g., “My parents let me do the things I think are important”); a negative dimension including parenting styles of rejection (e.g., “Nothing I do is good enough for my parents”), chaos (e.g., “My parents get mad at me with no warning”) and coercion (e.g., “My parents are always telling me what to do”). Each parenting style is composed of four questions with the response scale for each question ranging from not at all true (0) to very true (3). The adolescents were asked to consider both caregivers when answering the PASCQ (Skinner et al. 2005).

A positive summation index of only positive parenting styles (PASCQ^pos^, 12 items) was then created (0–36 points). The internal consistency for the PASCQ^pos^ demonstrated a Cronbach’s *α* = 0.832. For a further description of the evaluation and psychometric properties of the PASCQ, please see (Keijser et al. 2020a).

#### Genotyping

Genetic information for *FKBP5* was assessed during 2012 (Wave I) when participants were either 13 or 15 years old. Saliva samples for genotyping were collected using the Oragene^®^ DNA self-collection kit (Ottawa, Ontario, Canada) and extracted in accordance with the manufacturer’s guidelines. Genotyping was performed using a fluorescence-based competitive allele specific PCR (KASPar) assay (KBioscience^®^). Allele discrimination was done using SNPviewer^®^. The genotype calling was performed blind to psychosocial data. Genotypes were coded assuming an additive function and based on minor allele count: 0 = homozygous for the major allele, 1 = heterozygous and 2 = homozygous for the minor allele. The Hardy–Weinberg analyses showed that all SNPs were within the equilibrium (Table 1).

**Table 1:** Characteristics of the study sample, and Mann–Whitney *U* test for sex differences

Study variables	Total	Men	Women	Sex differences		
	M (SD)	M (SD)	M (SD)	*U*	*Z*	*p*
ELS (0–10)	2.86 (1.927)	2.76 (1.956)	2.93 (1.909)	109,173.000	– 2.016	0.044
Positive parenting (0–36)	28.76 (4.906)	28.42 (4.941)	28.97 (4.878)	109,865.500	– 1.816	0.069
Depressive symptoms Wave II (0–9)	2.82 (2.568)	1.81 (2.141)	3.41(2.613)	74,346.500	– 9.922	< 0.001
Depressive symptoms Wave III (0–9)	3.07 (2.805)	2.22 (2.583)	3.57 (2.812)	83,730.500	– 7.796	< 0.001
Number of participants	1006	372	634	–	–	–
Grouping variable sex: male = 0, female = 1						

*FKBP5* SNPs	*N* (%)			MAF	HWE *p*	Molecular consequence	Chromosome location
	Homozygous major	Heterozygous	Homozygous minor				
FKBP5 SNPs and haplotype characteristics							
*rs1360780*	CC 485 (58.1)	TC 391 (41.7)	TT 61 (6.5)	0.27	0.131	Intron variant	6:35639794
*rs3800373*	TT 528 (56.5)	TG 351 (37.6)	GG 55 (5.9)	0.25	0.740	3 prime UTR variant	6:35574699
*rs4713916*	GG 475 (51.0)	GA 382 (41.0)	AA 74 (7.9)	0.28	0.818	Intron variant	6:35702206
*rs7748266*	CC 670 (71.7)	TC 249 (26.7)	TT 15 (1.6)	0.15	0.133	Intron variant	6:35624967
*rs9296158*	GG 489 (52.5)	GA 382 (41.0)	AA 61 (6.5)	0.27	0.236	Intron variant	6:35599305
*rs9394309*	AA 462 (49.6)	GA 394 (49.6)	GG 75 (8.1)	0.29	0.479	Intron variant	6:35654004
*rs9470080*	CC 416 (44.8)	TC 430 (46.3)	TT 82 (8.8)	0.32	0.061	Intron variant	6:35678658
Haplotype		*N* (%)				–	–
TGCCACG:TGCCACG		403 (43.0%)					
TGCCACG:minor haplotype		438 (46.7%)					
Minor haplotype:minor haplotype		96 (10.2%)					

*FKBP5 SNPs* *FKBP5* polymorphism receptors, *HWE p* Hardy–Weinberg equilibrium probability, *MAF* minor allele frequency, *rs* reference SNP

Seven SNPs within the *FKBP5* gene were shortlisted, with the following minor alleles (MAF): the T allele of *rs1360780*, the G allele of *rs3800373*, the A allele of *rs4713916*, the T allele of *rs7748266*, the A allele of *rs9296158*, the G allele of *rs9394309* and the T allele of *rs9470080* (Table 1). The haplotype comprising the seven *SNPs* was categorized into: (1) only major alleles (no presence of minor alleles, defined as *FKBP5* low risk), (2) presence of at least one minor allele (1–6 minor alleles present; defined as *FKBP5* intermediate risk) and (3) haplotypes containing at least one minor allele in each haplotype (*FKBP5* high risk) (Isaksson et al. 2016). *FKBP5* haplotype low risk accounted for 43.0% of the participants, intermediate risk 46.7% of the participants, and high risk 10.2% of the participants (Table 1). For further description of the genotyping and haplotype procedures, please see (Isaksson et al. 2016).

### Analytic plan

All analyses and graph constructions were completed using the Statistical Package for Social Science (IBM SPSS Statistics for Windows, Version 26.0; Armonk, NY, USA). The PROCESS macro for SPSS version 3.4.1 was used to test and probe interaction effects (Hayes 2018).

For the consideration of significance through all analyses, a two-sided *p* value of 0.05 was considered significant (Fleiss 1986) in terms of recommendations regarding cG × E analyses (Dick et al. 2015). G*power was used to calculate needed effect size (Faul et al. 2009).

To test the hypothesis that missing values were missing completely at random, (Little 1988) test of missing completely at random (MCAR) was performed. The internal consistency regarding DSRS and PASCQ^pos^ were measured through Cronbach’s *α* with a cut-off of 0.7 for adequate consistency (George and Mallery 2016). The Kolmogorov–Smirnov test was used to test normality in the environmental variables, while the Hardy–Weinberg equilibrium was calculated by allele frequencies through an online spreadsheet. A partial correlation was used to explore the relationship between the study variables while adjusting for previous reports of depressive symptoms, sex and age to examine the multicollinearity in the data (Supplementary Table 1).

### Model specification

The main effects of ELS, PASCQ^pos^, each *FKBP5* SNP, and the haplotype on Wave III depressive symptoms, was analysed by multivariate linear regressions analyses by SPSS (Supplementary Table 2).

The three-way interaction analyses were conducted by an estimation of how depressive symptoms varied dependent on *FKBP5* SNPs or haplotype, ELS and PASCQ^pos^. The analyses were conducted using the PROCESS macro (moderated moderation in model 3) (Hayes 2018) and standardized beta (*β*) was assessed through multivariate linear regressions analyses by SPSS (Table 2).

**Table 2:** Regression estimates by *FKBP5* SNPs, ELS and positive parenting in relation to depressive symptoms during young adulthood

Model number	Regression estimates							Model evaluation		
	*β*	*B*	SE (HC3)	*t*	*p*	*∆R*^2^	*F*	*F*(*df 1*, *df 2*)	*R*^2^	*p*
1										
*rs1360780*	0.038	3.506	1.592	2.203	.028			22.978 (10, 907)	.198	< .001
ELS	0.106	0.270	0.435	0.620	0.537					
PASCQ^pos^	− 0.89	− 0.024	0.048	− 0.497	0.619					
rs1360780 × ELS	0.023	− 0.939	0.476	− 1.975	0.049					
*rs1360780* × PASCQ^pos^	− 0.026	− 0.121	0.054	− 2.253	0.024					
ELS × PASCQ^pos^	0.046	− 0.005	0.015	− 0.330	0.742					
*rs1360780* × ELS × PASCQ^pos^	0.069	0.035	0.016	2.111	0.035	0.004	4.455*			
Depressive symptoms Wave II	0.305	0.334	0.040	8.246	< 0.001					
Age	− 0.020	− 0.091	0.168	− 0.540	0.590					
Sex	0.144	0.836	0.187	4.469	< 0.001					
3										
rs4713916	0.076	1.899	1.636	1.161	0.246			23.742 (10, 901)	0.201	< 0.001
ELS	0.102	0.317	0.403	0.787	0.432					
PASCQ^pos^	− 0.089	− 0.049	0.046	− 1.055	0.292					
rs4713916 × ELS	0.033	− 0.888	0.459	− 1.933	0.053					
*rs4713916* × PASCQ^pos^	0.039	− 0.062	0.056	− 1.113	0.266					
ELS × PASCQ^pos^	0.038	− 0.007	0.014	− 0.530	0.596					
*rs4713916* × ELS × PASCQ^pos^	0.069	0.034	0.016	2.098	0.036	0.004	4.400*			
Depressive symptoms Wave II	0.311	0.340	0.040	8.472	< 0.001					
Age	− 0.019	− 0.092	0.169	− 0.544	0.586					
Sex	0.148	0.860	0.187	4.599	< 0.001					
4										
rs7748266	0.094	5.832	2.050	2.845	0.005			23.937 (10, 905)	0.206	< 0.001
ELS	0.108	0.323	0.387	0.837	0.403					
PASCQ^pos^	− 0.106	− 0.036	0.043	− 0.841	0.401					
rs7748266 × ELS	0.008	− 1.619	0.573	− 2.824	0.005					
*rs7748266* × PASCQ^pos^	− 0.022	− 0.186	0.069	− 2.707	0.007					
ELS × PASCQ^pos^	0.036	− 0.006	0.013	− 0.454	0.650					
*rs7748266* × ELS × PASCQ^pos^	0.105	0.057	0.020	2.900	0.004	0.009	8.411**			
Depressive symptoms Wave II	0.299	0.327	0.041	8.062	< 0.001					
Age	− 0.021	− 0.099	0.167	− 0.589	0.556					
Sex	0.141	0.817	0.185	4.408	< 0.001					
5										
rs9394309	0.067	2.281	1.634	1.396	0.163			23.087 (10, 901)	0.200	< 0.001
ELS	0.102	0.355	0.410	0.865	0.387					
PASCQ^pos^	− 0.091	− 0.040	0.048	− 0.848	0.396					
rs9394309 × ELS	0.031	− 0.926	0.463	− 2.000	0.046					
*rs9394309* × PASCQ^pos^	0.027	− 0.076	0.056	− 1.372	0.171					
ELS × PASCQ^pos^	0.039	− 0.009	0.014	− 0.608	0.543					
*rs9394309* × ELS × PASCQ^pos^	0.071	0.035	0.016	2.160	0.031	0.005	4.666**			
Depressive symptoms Wave II	0.311	0.339	0.040	8.439	< 0.001					
Age	− 0.021	− 0.094	0.169	− 0.557	0.578					
Sex	0.144	0.836	0.186	4.485	< 0.001					

Models adjusted for: depressive symptoms Wave II, sex (men = 0 and women = 1) and age (1997 = 1, 1999 = 0)

*β* standardized regression coefficient by multiple linear regression, *b* unstandardized regression coefficient, *SE (HC3)* heteroscedasticity-consistent standard error estimator, *ΔR*^*2*^ *R*^2^ change due to interaction, *PASCQ*^*pos*^ Parents as Social Context Questionnaire positive summation index, *ELS* Early Life Stress, *FKBP5 SNPs* *FKBP5* polymorphism receptors, *rs* reference SNP

*p < 0.05, **p < 0.01

To assess the potential three-way interaction effects, an evaluation through eight different models was made. Each model was consistent with either the haplotype or one of the *FKBP5* SNPs (*rs1360780*, *rs3800373*, *rs4713916*, *rs7748266*, *rs9296158*, *rs9394309* and *rs9470080*) as independent variable, ELS and PASCQ^pos^ as moderators and depressive symptoms among young adults as the dependent variable. All variables were modelled as discrete, and all interaction models were adjusted for the covariates sex (men = 0 and women = 1) and age (coded as born in 1997 = 1 or 1999 = 0) and previous reports of depressive symptoms (Wave II).

**Fig. 1:**
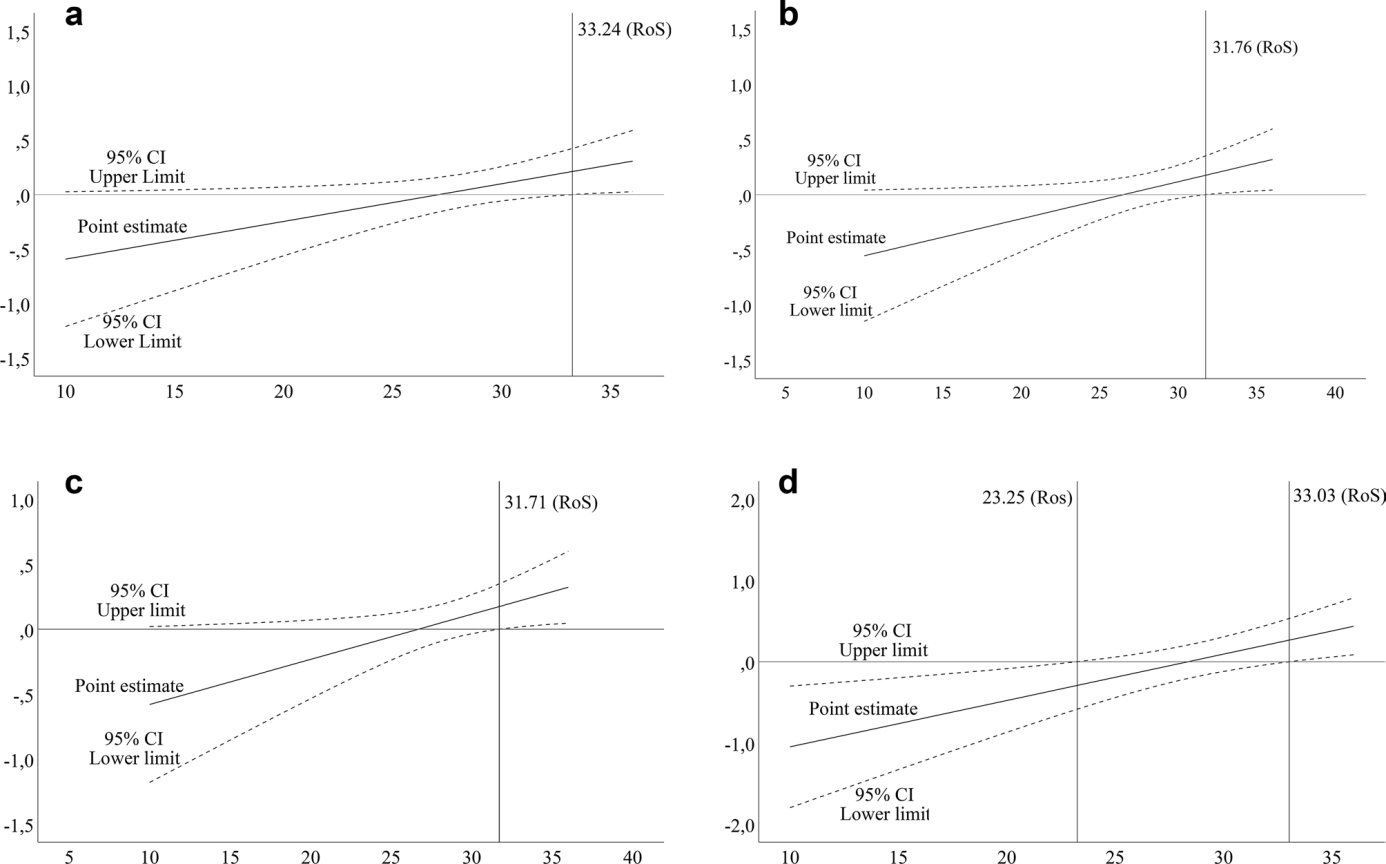
**a** Conditional *FKBP5* single-nucleotide polymorphism (SNP) rs1360780 × early life stress (ELS) interaction at values of positive parenting with 95% confidence interval (CI). The vertical line represents the limit of the Johnson–Neyman region of significance. **b** Conditional *FKBP5* SNP *rs4713916* × ELS interaction at values of positive parenting with 95% CI. The vertical line represents the limit of the Johnson Neyman region of significance. **c** Conditional *FKBP5* SNP *rs9394309* × ELS interaction at values of positive parenting with 95% CI. The vertical line represents the limit of the Johnson–Neyman region of significance. **d** Conditional *FKBP5* SNP *rs7748266* × ELS interaction at values of positive parenting with 95% CI. The vertical lines represent the limits of the Johnson–Neyman regions of significance. All figures were adjusted for sex, age, and previous depressive symptom scores

**Fig. 2:**
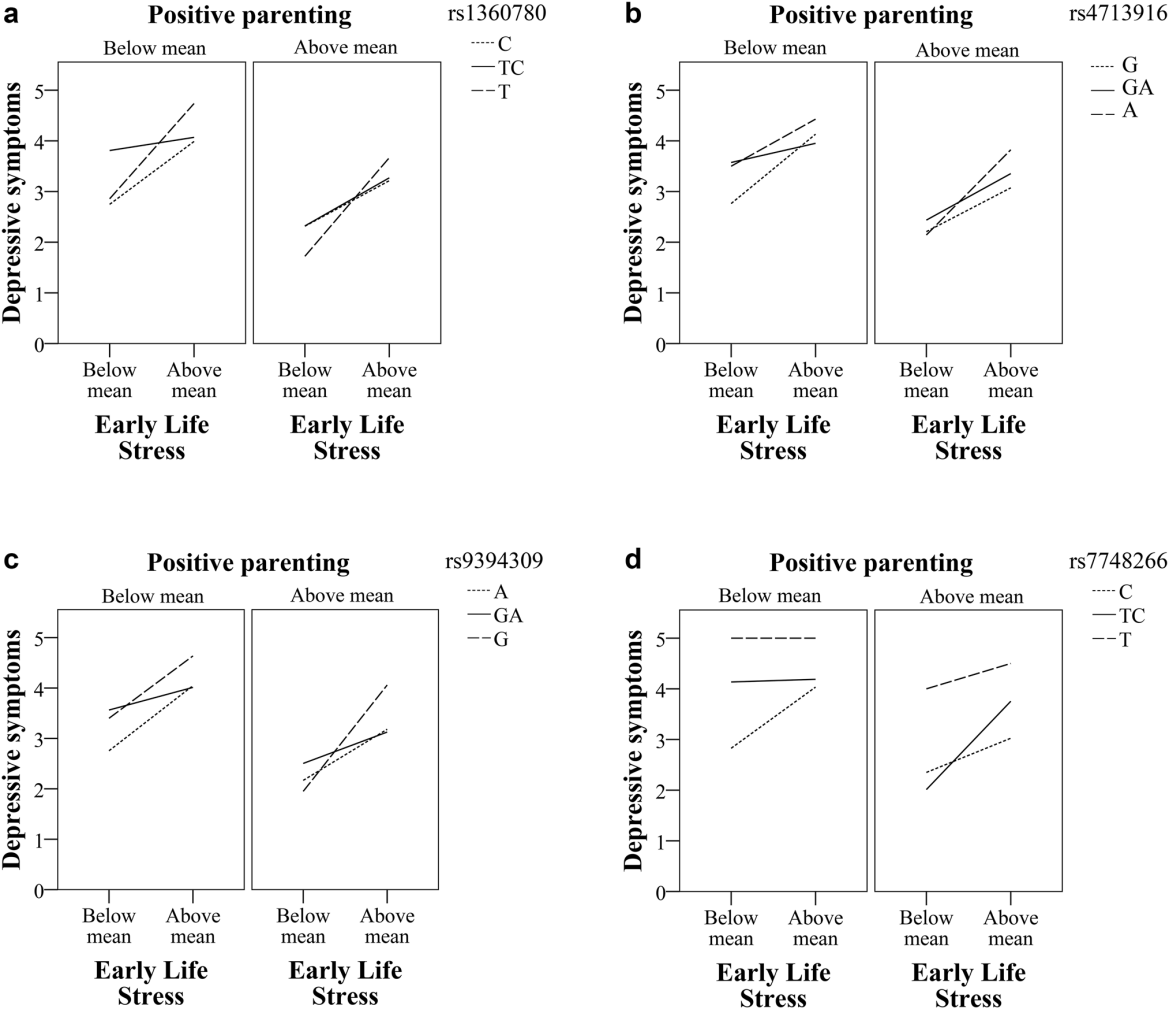
**a** Interaction effect of *FKBP5* SNP *rs1360780* and ELS on depressive symptoms divided by high and low positive parenting. **b** Interaction effect of *FKBP5* SNP *rs4713916* and ELS on depressive symptoms divided by high and low positive parenting. **c** Interaction effect of *FKBP5* SNP *rs9394309* and ELS on depressive symptoms divided by high and low positive parenting. **d** Interaction effect of *FKBP5* SNP *rs7748266* and ELS on depressive symptoms divided by high and low positive parenting. All figures were adjusted for sex, age and previous depressive symptom scores

To probe the interactions of cG × E in the context of the diathesis stress model and differential susceptibility theory, the Johnson–Neyman technique (Johnson and Neyman 1936; Lazar and Zerbe 2011; Roisman et al. 2012) was used for all significant models. In particular, the regions of significance (RoS) of the *FKBP5* SNPs × ELS at values of the PASCQ^pos^ were evaluated (Fig. 1). A single RoS value indicates support for a diathesis stress effect and two RoS values indicate support for differential susceptibility effects. The RoS index was restricted to a range of interest ± 2 SD from the mean of the PASCQ^pos^, as recommended (Roisman et al. 2012). As a last step, all significant three-way interaction effects were probed in linear graphs to visualize the data (Fig. 2). The graphs were illustrated based on mean values of the PASCQ^pos^ and ELS (mean values are presented in Table 1) and categorical allele type (allele frequency is presented in Table 1).

### Data evaluation

The total proportions of missing values on the DSRS and PASCQ^pos^ items were 0.006% and 0.34%, respectively. The results from (Little 1988) test of MCAR for the DSRS (*χ*^2^ = 21.222, *df* = 14, *p* = 0.096) showed that missing values were missing completely at random, whereas the results for the PASCQ^pos^ (*χ*^2^ = 161.604, *df* = 133, *p* = 0.046) showed that the missing values in the data were not missing completely at random. A decision to exclude 28 individuals due to missing data in the study variables was made after consulting the Mahalanobis analysis with the cut-off set to 13.82 (*χ*^2^ table, *df* = 2, *p* < 0.001). After exclusion, 1006 participants remained in the sample.

Partial correlation was used to explore the relationship between the study variables while adjusting for age and sex to rule out multicollinearity. A significant correlation was seen between ELS and *rs7748266* (*r* = 0.074, *p* = 0.029) indicating collinearity (Supplementary Table 1). Tests to see if the data met the assumption of collinearity indicated that multicollinearity was not a concern (ELS, tolerance = 0.925, variance inflation factor = 1.081; *rs7748266*, tolerance = 0.949, variance inflation factor = 1.054). No other independent variables were significantly correlated, and multicollinearity was not a concern (Supplementary Table 1).

The Kolmogorov–Smirnov test indicated that the environmental study variables did not follow a normal distribution; DSRS *D* (1018) = 0.156, *p* < 0.001, ELS *D* (1018) = 0.216, *p* < 0.001 and PASCQ^pos^ *D* (1018) = 0.123, *p* < 0.001. The normal PP plot of regression standardized residual was used to evaluate the linearity and homogeneity (data not shown). Heteroscedasticity was evaluated through a scatterplot with standardized residuals (data not shown). Because the assumption of equal variances was violated, the heteroscedasticity-consistent (HC) standard error estimator HC3–Davidson–MacKinnon was used (Darlington and Hayes 2017; White 1980).

## Results

The characteristics and genotype frequencies of the study sample are shown in Table 1. The study variables ELS, PASCQ^pos^, the *FKBP5* SNPs (*rs1360780*, *rs3800373*, *rs4713916*, *rs7748266*, *rs9296158*, *rs9394309* and *rs9470080*) and the haplotype were first tested as unconditional main effects on depressive symptoms in multivariate linear regression models. Significant main effects were found for *FKBP5* SNPs *rs4713916*, *rs7748266*, *rs9394309*, ELS and PASCQ^pos^ (Supplementary Table 2). The study variables ELS, PASCQ^pos^, the *FKBP5* SNPs (*rs1360780*, *rs3800373*, *rs4713916*, *rs7748266*, *rs9296158*, *rs9394309* and *rs9470080*) and the haplotype were then tested as a three-way interaction in the PROCESS macro (moderated moderation in model 3) (Hayes 2018). The significant models are presented in Table 2 and non-significant models are presented in Supplementary Table 3.

### Model evaluation

The three-way interactions of ELS, PASCQ^pos^ and each *FKBP5* SNP (i.e., *rs1360780*, *rs4713916*, *rs7748266* and *rs9394309*) on depressive symptoms among young adults, adjusted for age, sex and previous reports of depressive symptoms were significant (Table 2). The models each accounted for approximately 20% individually, including all main and interaction effects and accounted for depressive symptoms among young adults (Table 2).

### Evaluation of *FKBP5* SNPs

#### rs1360780

The three-way interaction of *rs1360780* × ELS × PASCQ^pos^ significantly accounted for approximately 0.4% of the variance in depressive symptoms among young adults (Table 2). The conditional effects of *rs1360780* × ELS at values of PASCQ^pos^ indicated significant effects on depressive symptoms at higher levels of positive parenting, *F*(1, 907) = 4.019, *p* = 0.045. A single RoS value on the PASCQ^pos^ corresponded to a score of 1SD above the mean, *t*(907) = 1.963, *p* = 0.05, suggesting a difference in the effect of ELS between C, TC and T carriers among those that estimated PASCQ^pos^ scores of ≥ 33 points (Fig. 1a). The three-way interaction of *rs1360780* × ELS × PASCQ^pos^ was then visualized in a graph, indicating that the T allele carriers, homozygous minor, reported higher levels of depressive symptoms when high levels of ELS were present, yet the least depressive symptoms as lower levels of ELS were present under the influence of higher levels of positive parenting in comparison with TC and C carriers (Fig. 2a).

#### rs4713916

The three-way interaction of *rs4713916* × ELS × PASCQ^pos^ accounted for approximately 0.4% of the variance in depressive symptoms among young adults (Table 2). The conditional effects of *rs4713916* × ELS at values of PASCQ^pos^ indicated significant effects on depressive symptoms at the higher end of positive parenting, *F*(1, 901) = 4.714, *p* = 0.030. A single RoS value on the PASCQ^pos^ corresponded to a score of 1SD above the mean*, t*(901) = 1.963, *p* = 0.050, suggesting a difference in the effect of ELS between G, GA and A carriers among those that estimated PASCQ^pos^ scores of at least 32 points (Fig. 1b).

The three-way interaction of *rs4713916* × ELS × PASCQ^pos^ was then visualized in a graph, indicating that the A allele carriers, homozygous minor, reported higher levels of depressive symptoms when high levels of ELS were present; however, the least depressive symptoms at lower levels of ELS were present under the influence of higher levels of positive parenting in comparison with GA and G carriers (Fig. 2b).

#### rs9394309

The three-way interaction of *rs9394309* × ELS × PASCQ^pos^ accounted for approximately 0.5% of the variance in depressive symptoms among young adults (Table 2). The conditional effect of *rs9394309* × ELS at high values of PASCQ^pos^ indicated significant effects on depressive symptoms at the higher end of positive parenting, *F*(1, 901) = 4.826, *p* = 0.028. A single RoS value on the PASCQ^pos^ corresponded to a score of 1SD above the mean *t*(901) = 1.963, *p* = 0.050, suggesting a difference in the effect of ELS between A, AG and G carriers among those that estimated PASCQ^pos^ scores of at least 32 points (Fig. 1c).

The three-way interaction of *rs9394309* × ELS × PASCQ^pos^ was visualized in a graph, indicating that the G allele carriers, homozygous minor, reported higher levels of depressive symptoms when high levels of ELS were present, yet the least depressive symptoms as lower levels of ELS were present under the influence of higher levels of positive parenting in comparison with GA and A carriers (Fig. 2c).

#### rs7748266

The three-way interaction of *rs7748266* × ELS × PASCQ^pos^ significantly accounted for approximately 0.9% of the variance in depressive symptoms among young adults (Table2). The conditional effect of rs7748266 × ELS at values of PASCQ^pos^ indicated significant effects on depressive symptoms of positive parenting, *F*(1, 905) = 4.419, *p* = 0.036. Two RoS values on the PASCQ^pos^ corresponded to scores of approximately 1 SD below the mean, *t*(905) = – 1.963, *p* = 0.050, and approximately 1 SD above the mean, *t*(905) = 1.963, *p* = 0.050, suggesting a difference in the effect of ELS between C, TC and T carriers among those that estimated PASCQ^pos^ scores of a maximum of 23 points or a minimum of 33 points (Fig. 1d).

The three-way interaction of *rs7748266* × ELS × PASCQ^pos^ was visualized in a graph, indicating that the T allele carriers, homozygous minor, reported higher levels of depressive symptoms overall, independent of environment, in comparison with TC and C carriers (Fig. 2d).

For a complete evaluation of all terms, please consult Table 2, where the full models are presented.

## Discussion

The present study found three-way interaction effects for four *FKBP5* SNPs (*rs1360780*, *rs4713916*, *rs7748266* and *rs9394309*) with ELS and positive parenting in relation to the variance in depressive symptoms among young adults. Furthermore, the present study provided findings for the diathesis stress pattern of interactions regarding *rs1360780*, *rs4713916* and *rs9394309*, and differential susceptibility patterns of interaction for *rs7748266*.

Four out of seven *FKBP5* SNPs (*rs1360780*, *rs4713916*, *rs9394309* and *rs7748266)* presented significant effects in their models, with positive parenting style and ELS on depressive symptoms, indicating that not all *FKBP5* SNPs had an effect with environmental factors in the present sample. This might also explain the lack of findings regarding the haplotype. The models individually accounted for approximately 20% of the variance in depressive symptoms among young adults, including all main and interaction effects. Because depression emerges from different factors and the pathways to depression are complex (Kaufman et al. 2001; Sullivan et al. 2000), an overall variance of approximately 20% in depression dependent on the study variables is satisfactory given that tests were made prior to the analyses for model fit.

The significant interactions were further probed in an approach where the secondary moderator (positive parenting style) was selected with the goal of ascertaining whether ELS moderated the *FKBP5* SNP (*rs1360780*, *rs4713916*, *rs9394309*, or *rs7748266*, i.e., cG) effect on depressive symptoms conditional on values of positive parenting style. This required an estimation of the conditional effect of the cG × ELS interaction given positive parenting style as well as an inferential test for this conditional interaction at positive parenting style values. All transition points for a significant effect on positive parenting were ${\mathrm{M}}_{\pm 1 \mathrm{SD}}$. There was a statistically significant difference in the effect of ELS between the alleles of the *FKBP5* SNPs (*rs1360780*, *rs4713916*, *rs9394309* and *rs7748266*) on depressive symptoms among young adults (Fig. 1a–d). The complete understanding of these effects is complex; however, the findings suggest that there is a moderating effect of positive parenting style in each significant model. ELS seemed to have a robust effect with the different SNPs in each model, with significant beta values ranging from 0.88 to 1.6, although these were not significant as a main effect in any model.

One aspect of these findings could be that the positive environment was preceded by a negative environment (Saltz et al. 2018), that is, the negative environment might have a greater impact than the positive. Another aspect may be that of the cognitive diathesis–stress model that proposes a tendency among vulnerable individuals of evaluating negative exposures, or their consequences, worse than others and thereby be more likely to develop depressive symptoms than individuals without such cognitive tendencies (Abramson et al. 1989; Hammen 1991).

For plasticity to transpire, a reaction to stimuli in the environment and a response to it by an adjustment in the phenotype is needed. The differences in the responsiveness and/or the sensitivity among the genotypes defines the cG × E interaction (Saltz et al. 2018). The understanding of such sensitivity and responsiveness in the context of cG × E is conceptual when interpreting and understanding the variation in different or new environments (Saltz et al. 2018).

Duncan and Keller (2011) have criticized the many positive cG × E findings reported in the psychiatric literature and suggested that such findings are consistent with the existence of publication bias among novel cG × E studies, making cG × E hypotheses appear more robust than they actually are. However, the non-significant findings of the study of well evaluated models by Pérez-Pérez et al. (2018), and the construction of theoretically based models in the present study, suggest this should not be the case here with *FKBP5*. Another aspect of cG × E studies is the choice of environmental measures. It is critical that the measures are reliable, with empirical precedents and theoretically plausible (Dick et al. 2015). It has been argued that cG × E studies ought to identify novel environmental factors to improve the understanding of the etiologic role of different factors for health issues (Hutter et al. 2013) and that a key concept in such studies is the assessment of the environmental factor (Thomas 2010). Environmental factors are complex and multidimensional and can lead to unpredicted biases inducing spurious interactions (Thomas 2010). Therefore, a sound approach may be to limit the number of different variables in the model (Nilsson et al. 2018) to be better able to interpret the findings.

Since the 1980s, the family environment has been a known contributor to the onset and maintenance of mood disorders (Burbach and Borduin 1986; Gerlsma et al. 1990), with studies confirming that both the absence and presence of positive parenting (Chen et al. 2009; del Barrio et al. 2016; Keijser et al. 2020b; Schwartz et al. 2014; Schwartz et al. 2017) and excessive external stress (in particular, ELS) (Wang et al. 2020) have an effect on depression. The findings of significant main effects of the environmental factors ELS and PASCQ^pos^ on depressive symptoms among young adults in the present study were, therefore, expected. There is also a cumulative additive family risk of ELS when the combined effects of socio-economic difficulties (such as low parental income, unemployment and housing instability) and parental characteristics (such as mental and/or physical health) are considered (Patwardhan et al. 2017). The cumulative additive risks of ELS are not measured in most of the conceptualized cG × E models, which is called the predictor–intersection problem (Nilsson et al. 2018). A sound approach to address the predictor–intersection problem may be to use ELS indexes that consider several types of negative environmental factors (Nilsson et al. 2018). The present study approached this problem using a summarizing question to assess the total subjective impact of the reported stressful events on the child’s experienced stress during childhood.

Another important aspect of cG × E studies is the developmental timing for measuring the environmental variables because social and biological impacts tend to vary as a function of developmental stages (Dick et al. 2015). The collection of ELS reports during childhood (however, retrospectively) and PASCQ^pos^ during adolescence are preferable as both stages are known to be sensitive periods in which individual experiences form traits to a greater extent than they would during other periods of life (Fawcett and Frankenhuis 2015). Parenting styles are complex and include different kind of behaviours that influence child outcomes. Thereby, isolating only one behaviour may be misleading (Darling 1999). The concept of parenting styles is intended to capture the broad perspective of parenting with its normal variation (Baumrind 1991) and with transitions between different subtypes of parenting styles between parents and adolescents (Zhang et al. 2017). Moreover, perceived parenting styles reported by adolescents might be influenced by different factors, such as genetic aspects (Moffitt 2005). However, the subjective understanding of parenting styles might enable further knowledge of the mechanisms by which parenting styles influence adolescent outcomes (Powers et al. 1994).

The overall associations between *FKBP5* and depression confirm previous findings (Binder 2009; Lavebratt et al. 2010; Normann and Buttenschøn 2019; Scheuer et al. 2015; Wang et al. 2018; Zannas and Binder 2014; Zimmermann et al. 2011). In our study, reports of higher levels of positive parenting having an overall decreasing effect on depressive symptoms independent of allele type and in the presence of previous lower or higher exposure to ELS compared with reports of lower levels of positive parenting are consistent with suggestions that a positive environment can reduce the long-term negative effects of stress on altered HPA function (Morley-Fletcher et al. 2003). These effects are also in line with previous findings of a longitudinal effect of positive parenting on decreased depressive symptom severity that is consistent over time (del Barrio et al. 2016).

Our findings regarding the diathesis stress or differential susceptibility effects partly confirm the previous literature. Researchers have presented results indicating support for the *FKBP5* in the diathesis stress and/or differential susceptibility theorem without performing statistical tests to evaluate the form of these interactions (Bevilacqua 2012; Scheuer et al. 2015; VanZomeren-Dohm et al. 2015; Xie et al. 2010; Zimmermann et al. 2011). Only one previous study of *FKBP5* has evaluated both positive and negative aspects regarding differential susceptibility in a cross-sectional study; that is, Pérez-Pérez et al. (2018) found no significant interaction effects in relation to depressive symptoms. However, support for differential susceptibility interactions was found for anxiety and neuroticism (Pérez-Pérez et al. 2018). The importance of cG × E and its role in the diagnosis and severity of several psychiatric disorders, such as depression, are acknowledged because individual differences in the susceptibility to environmental factors may be under the influence of candidate genes (Musci et al. 2019). Notably, although no statistically significant differential susceptibility effects were found using the Johnson–Neyman technique for the *FKBP5* SNPs (*rs1360780*, *rs4713916* and *rs9394309*), the patterns shown in the graphs, plotting positive parenting and ELS dichotomously (above and below the mean) and the genotypes as categorical values, indicated differential susceptibility patterns for the homozygous minor alleles (T, A and G, respectively) (Fig. 2a–c). Although these patterns are certainly intriguing, no statistically significant evidence for susceptibility properties of these alleles were found. As the limited sample in the present study might involve problems with statistical power, we thereby encourage further evaluation of possible susceptibility properties of *FKPB5* in larger, more diverse samples.

Epigenetic aspects of cG × E findings may be of importance for future research. Previous research states that the epigenetic aspects of *FKBP5* are particularly interesting (Klengel and Binder 2015; Zannas and Binder 2014). Klengel et al. (2013) found that *FKBP5* mediated the interaction of genetic and environmental effects on stress-related psychiatric disorders in adulthood by DNA methylation in functional glucocorticoid-response elements of *FKBP5*. Weder et al. (2014) found differences in *FKBP5* methylation between children exposed and non-exposed to maltreatment in relation to depression. Park et al. (2019) stated that epigenetic changes in a glucocorticoid signalling gene, such as *FKBP5*, should be one of the most promising and beneficial aims for future research. Regarding epigenetic changes in depression, Sun et al. (2013) proposed in their review that defining the complex architecture of genes that show altered patterns of methylation and several chromatin modifications in brain regions can explain how epigenetic mechanisms control the robust changes in gene expression and regulation that influence the development and treatment of depression.

### Strengths and limitations

The inclusion of both SNPs and haplotype in the analyses should be seen as a strength, with the latter amplifying the power in genetic research to detect possible associations (Crawford and Nickerson 2005). The lack of findings regarding the haplotype may indicate that not all genetic variants have an effect. This was further supported by four of the seven SNPs showing significant effects in their separate models. The design of the study offers a useful model evaluation for future research on cG × E interaction including *FKBP5* in a general population, despite the null findings for the haplotype.

The longitudinal approach brings an interesting aspect to this study where stress during childhood and parenting style during adolescence may affect depressive symptoms during early adulthood. However, further research is needed to establish the findings. A larger sample, a clinical sample or even a wider time range would be of interest.

The accuracy of a summarizing question instead of multiple items to measure ELS must be addressed. The negative environment was not used as a rating scale of the frequency of ELS but as a measurement of the caregiver’s subjective evaluation of the impact of ELS exposure on the level of experienced stress by the child during childhood. As mentioned earlier, the stimuli of the environment were essential in the cG × E interaction in the present study and the specific source of stress was less important. The wider context of total stress exposure that stimulates the HPA axis and causes long-term stress during childhood was targeted by this measurement in the present study (Nilsson et al. 2018).

Retrospective self-reports induce the risk for report bias, which is to be considered a limitation in the present study. Nevertheless, all measurements used showed good reliability and had been evaluated prior to this study (Hero 2013; Keijser et al. 2020a; Skinner et al. 2005; Svanborg and Ekselius 2003; Tabachnick and Fidell 2007).

Biological data were not collected from the parents in the present study, but could have added another dimension to the findings.

The present study did not control for psychopharmacology or therapy treatment for depression, although these aspects could be confounders in terms of increases in depressive symptoms. cG × E studies are sensitive to confounders, even if some are considered mandatory to control for in models with interaction terms (Zannas and Binder 2014). The inclusion of several predictors could have decreased the power further, and the results would have been more difficult to interpret.

## Conclusion

The findings of the present study indicate an interaction of *FKBP5* SNPs *rs1360780*, *rs4713916*, *rs9394309* and *rs7748266* with environmental factors in relation to the variance in depressive symptoms in young adulthood. The effects of the *FKBP5* alleles on depressive symptoms seemed to be moderated by ELS during childhood and positive parenting during adolescence. The evaluations made in this study may contribute to further research on the role of *FKBP5* in the development of depressive symptoms. Researchers involved in the research field of development of mental health, cG × E studies and clinicians that are working with family treatment might benefit from these findings.

## Supplementary Information

Below is the link to the electronic supplementary material.

Supplementary file1 (DOCX 47 KB)

## Data Availability

Not applicable.
