# Supplementary material for: Three-way interaction effects of early life stress, positive parenting and FKBP5 in the development of depressive symptoms in a general population
Source: J Neural Transm (Vienna). 2021 Aug 22;128(9):1409–24. doi: 10.1007/s00702-021-02405-0 (PMC8423649; doi:10.1007/s00702-021-02405-0)
Supplement: Supplementary file 1 — Supplementary file1 (DOCX 47 KB) [file 702_2021_2405_MOESM1_ESM.docx]

| **Supplementary Table 1.** Partial correlation analysis of the study variables *FKBP5* SNP, *FKBP5* Haplotype, ELS, PASCQ^pos^ and depressive symptoms | | | | | | | | | | | |
| --- | --- | --- | --- | --- | --- | --- | --- | --- | --- | --- | --- |
| Variables | Depressive symptoms | PASCQ^pos^ | ELS | rs1360780 | rs3800373 | rs4713916 | rs7748266 | rs9296158 | rs9394309 | rs9470080 | Haplotype |
| Depressive symptoms | - | -0.105^**^ | 0.110^***^ | 0.049^ns^ | 0.031^ns^ | 0.089^**^ | 0.101^**^ | 0.033^ns^ | 0.080^*^ | 0.067^*^ | 0.065^ns^ |
| PASCQ^pos^ |  | - | -0.046^ns^ | -0.036^ns^ | -0.028^ns^ | -0.041^ns^ | -0.047^ns^ | -0.035^ns^ | -0.046^ns^ | -0.033^ns^ | -0.029^ns^ |
| ELS |  |  | - | 0.040^ns^ | 0.028^ns^ | 0.057 ^ns^ | 0.074^**^ | 0.043^ns^ | 0.045^ns^ | 0.061^ns^ | 0.063^ns^ |
| rs1360780 | |  |  | - | 0.928^***^ | 0.739^***^ | 0.656^***^ | 0.970^***^ | 0.813^***^ | 0.875^***^ | 0.851^***^ |
| rs3800373 | |  |  |  | - | 0.659^***^ | 0.554^***^ | 0.940^***^ | 0.735^***^ | 0.809^***^ | 0.796^***^ |
| rs4713916 | |  |  |  |  | - | 0.629^***^ | 0.709^***^ | 0.924^***^ | 0.864^***^ | 0.887^***^ |
| rs7748266 | |  |  |  |  |  | - | 0.651^***^ | 0.625^***^ | 0.577^***^ | 0.573^***^ |
| rs9296158 | |  |  |  |  |  |  | - | 0.783^***^ | 0.865^***^ | 0.847^***^ |
| rs9394309 | |  |  |  |  |  |  |  | - | 0.930^***^ | 0.886^***^ |
| rs9470080 | |  |  |  |  |  |  |  |  | - | 0.961^***^ |
| Haplotype |  |  |  |  |  |  |  |  |  |  | - |
| *Note*: Correlations adjusted for: Depressive symptoms WII, Age, and Sex; Depressive symptoms = Depressive symptoms WIII; ELS = Early life stress, PASCQ^pos^ = Parents as Social Context Questionnaire positive summation index: rs = Reference SNP; *FKBP5* SNP = *FKBP5* polymorphism receptor; ^*^*p* <0.05. ^**^*p* < 0.01. ^***^*p*<0.001. ^ns^ = non-significant | | | | | | | | | | | |

| **Supplementary Table 2.** Univariate regression analyses of the main effects of PASCQ^pos^, ELS and *FKBP5* in relation to depressive symptoms among young adults | | | | | | | |
| --- | --- | --- | --- | --- | --- | --- | --- |
|  |  | Linear univariate regression | | | | | |
| Dependent variable | Independent variables | *b* | *95% CI* | *SE* | *t* | *η_p_* | *p* |
| Depressive symptoms | ELS | .262 | –174–.351 | .045 | 5.803 | .032 | <.001 |
|  | PASCQ^pos^ | –.103 | –.138– –.069 | .018 | –5.831 | .003 | <.001 |
|  | *rs1360780* | .219 | –.076–.515 | .150 | 1.457 | .002 | .145 |
|  | *rs3800373* | .210 | –.089–.510 | .153 | 1.378 | .002 | .169 |
|  | *rs4713916* | .310 | .024–.596 | .146 | 2.125 | .005 | .034 |
|  | *rs7748266* | .524 | .156–.891 | .187 | 2.794 | .008 | .005 |
|  | *rs9296158* | .196 | –.100–.492 | .151 | 1.300 | .002 | .194 |
|  | *rs9394309* | .312 | .026–.597 | .145 | 2.145 | .005 | .032 |
|  | *rs9470080* | .264 | –.022–.549 | .145 | 1.815 | .004 | .070 |
|  | Haplotype | .233 | –.045–.511 | .142 | 1.645 | .003 | .100 |
| *Note*: *b* coefficient = unstandardized regression; CI = Confidence interval; *η_p_* = eta squared; *FKBP5* SNPs; PASCQ^pos^ – The Parents as Social Context Questionnaire positive summation index; rs = Reference SNP | | | | | | | |

| **Supplementary Table 3** Regression estimates by *FKBP5* SNPs, ELS and positive parenting in relation to depressive symptoms during young adulthood | | | | | | |
| --- | --- | --- | --- | --- | --- | --- |
| Model | Regression estimates | | | | | |
| *2* | *b* | *SE(HC3)* | *t* | *p* | *Δ R^2^* | *F* |
| *rs3800373* | 1.956 | 1.605 | 1.219 | .223 |  |  |
| ELS | –.107 | .390 | –.273 | .785 |  |  |
| PASCQ^pos^ | –.054 | .046 | –1.173 | .241 |  |  |
| rs3800373 × ELS | –.360 | .466 | –.773 | .440 |  |  |
| *rs3800373* × PASCQ^pos^ | –.073 | .054 | – 1.361 | .174 |  |  |
| ELS × PASCQ^pos^ | .007 | .013 | .559 | .576 |  |  |
| *rs3800373* × ELS × PASCQ^pos^ | .015 | .016 | .967 | .334 | .001 | .936 |
| *Depressive symptoms wave II* | .337 | .040 | 8.330 | <.001 |  |  |
| *Age* | -.070 | .081 | -.864 | .388 |  |  |
| *Sex* | .802 | .187 | 4.286 | <.001 |  |  |
| *5* | *b* | *SE(HC3)* | *t* | *p* | *Δ R^2^* | *F* |
| *rs9296158* | 2.725 | 1.619 | 1.683 | .093 |  |  |
| ELS | .118 | .454 | .261 | .794 |  |  |
| PASCQ^pos^ | –.039 | .050 | –.796 | .426 |  |  |
| *rs9296158* × ELS | –.709 | .483 | –1.469 | .142 |  |  |
| *rs9296158* × PASCQ^pos^ | –.099 | .055 | –1.803 | .072 |  |  |
| ELS × PASCQ^pos^ | .000 | .015 | .005 | .996 |  |  |
| *rs9296158* ×ELS × PASCQ^pos^ | .027 | .016 | 1.660 | .097 | .003 | 2.755 |
| *Depressive symptoms wave II* | .332 | .041 | 8.068 | <.001 |  |  |
| *Age* | -.062 | .081 | -.762 | .446 |  |  |
| *Sex* | .836 | .188 | 4.438 | <.001 |  |  |
| *7* | *b* | *SE(HC3)* | *t* | *p* | *Δ R^2^* | *F* |
| *rs9470080* | 1.776 | 1.630 | 1.089 | .276 |  |  |
| ELS | .176 | .458 | .384 | .701 |  |  |
| PASCQ^pos^ | –.052 | .050 | –1.044 | .297 |  |  |
| rs9470080 × ELS | –.667 | .486 | –1.372 | .170 |  |  |
| *rs9470080* × PASCQ^pos^ | –.063 | .055 | –1.145 | .253 |  |  |
| ELS × PASCQ^pos^ | –.003 | .016 | –.177 | .860 |  |  |
| *rs9470080* × ELS × PASCQ^pos^ | .026 | .017 | 1.577 | .115 | .003 | 2.486 |
| *Depressive symptoms wave II* | .331 | .040 | 8.196 | <.001 |  |  |
| *Age* | -.072 | .081 | -.885 | .376 |  |  |
| *Sex* | .875 | .188 | 4.646 | <.001 |  |  |
| *8* | *b* | *SE(HC3)* | *t* | *p* | *Δ R^2^* | *F* |
| Haplotype | 1.726 | 1.581 | 1.092 | .275 |  |  |
| ELS | .154 | .457 | .338 | .736 |  |  |
| PASCQ^pos^ | –.051 | .050 | –1.008 | .314 |  |  |
| Haplotype × ELS | –.618 | .460 | –1.343 | .180 |  |  |
| Haplotype × PASCQ^pos^ | –.062 | .054 | –1.153 | .249 |  |  |
| ELS × PASCQ^pos^ | –.002 | .016 | –.137 | .891 |  |  |
| Haplotype × ELS × PASCQ^pos^ | .025 | .016 | 1.553 | .121 | .002 | 2.411 |
| *Depressive symptoms wave II* | .336 | .040 | 8.359 | <.001 |  |  |
| *Age* | -.056 | .080 | -.690 | .491 |  |  |
| *Sex* | .861 | .187 | 4.615 | <.001 |  |  |
| *Note*: Models adjusted for: depressive symptoms wave II, Sex (men = 0 and women = 1) and age (1997 = 1, 1999 = 0); *b* coefficient = unstandardized regression coefficient; *Δ*R^2^ = R^2^ change due to interaction; PASCQ^pos^ = Parents as Social Context Questionnaire positive index; ELS = Early Life Stress; SE (HC3) = Heteroscedasticity- consistent standard error; *FKBP5* SNP = *FKBP5* polymorphism receptor, rs = Reference SNP | | | | | | |
